# Green Acerola (*Malpighia emarginata*) Extraction Optimization, Cellular Antioxidant Activity, and Spray Drying: Toward a Stable Vitamin C-Rich Powder

**DOI:** 10.1007/s11130-026-01536-7

**Published:** 2026-07-03

**Authors:** Richtier G. Cruz, Sébastien Dupont, Laurent Beney, Simone P. Lira, Thais Maria Ferreira de Souza Vieira

**Affiliations:** 1 Escola Superior de Agricultura Luiz de Queiroz, University of São Paulo, Av. Pádua Dias 11, Piracicaba, São Paulo, 13418-900 Brazil; 2 Faculty of Chemical Engineering, Federal University of Uberlandia, Campus Patos de Minas, Rua Vereador Chico Filgueira, 33, Patos de Minas, Minas Gerais 38702-178 Brazil; 3 Institut Agro, INRAE, UMR PAM, Université Bourgogne Europe, Dijon, F-21000 France

**Keywords:** Ascorbic acid, Response surface methodology, Oxidative stress, *Saccharomyces cerevisiae*, Encapsulation, Bioactive compounds

## Abstract

Green acerola (*Malpighia emarginata*) is among the richest natural sources of vitamin C and represents a promising raw material for natural antioxidant food ingredients. Extraction of ascorbic acid from whole green acerola fruits was optimized using response surface methodology, with solvent composition and temperature as independent variables. Pure water at 35 °C provided the highest ascorbic acid recovery (340 mg g⁻¹ dry material) and reducing power, alongside strong antioxidant activity by ABTS and DPPH assays. The biological relevance of the optimized extract was evaluated using *Saccharomyces cerevisiae* wild-type and *erg6*Δ mutant strains as a cellular antioxidant model under menadione-induced oxidative stress. In the presence of the extract (500 mg L⁻¹), cell viability was maintained for up to 5 h in the wild-type strain and 3 h in the *erg6*Δ mutant, compared to approximately 1 h in untreated controls. The extract was subsequently stabilized by spray drying with arabic gum as carrier, yielding a powder with water activity of 0.213 and no significant loss of antioxidant composition or activity. These results establish mild aqueous extraction combined with spray drying as an effective strategy for producing a stable, vitamin C-rich green acerola powder with demonstrated cellular antioxidant activity, suitable for food ingredient applications.

## Introduction

Ascorbic acid is one of the most important vitamins for human health and has numerous applications in the food and pharmaceutical industries. Beyond its role in preventing scurvy, ascorbic acid exhibits important antioxidant activity [1, 2]. Ascorbic acid is the vitamin with the highest industrial production volume, with approximately 50% directed to the pharmaceutical industry, 25% used as a food antioxidant, and the remainder consumed by the beverage and animal feed sectors. Current industrial production still relies largely on the Reichstein process, developed in the 1930s from D-glucose, which demands considerable amounts of organic solvents and generates environmental concerns [3]. The use of renewable raw materials and greener extraction processes therefore represents both a challenge and an opportunity for the sector.

Among tropical fruits, acerola (*Malpighia emarginata*) stands out for its exceptionally high vitamin C content, exceeding 4,000 mg *per* 100 g of fresh fruit, and strong antioxidant activity [4–6]. The green stage of the fruit is well established as a superior source of ascorbic acid compared to the ripe stage, with recognized potential for food and pharmaceutical applications [7, 8]. However, studies combining extraction optimization with cellular antioxidant validation are still lacking. This work optimized aqueous extraction of ascorbic acid from green acerola by response surface methodology, assessed antioxidant efficacy in vitro and in a *Saccharomyces cerevisiae* cellular model, and evaluated stabilization of the extract by spray drying.

## Materials and Methods

### Sample Collection and Acerola Extract Preparation

Samples of green acerola fruits were obtained from certified producers in the city of Junqueiropolis, Sao Paulo, Brazil. In order to minimize the degradation of bioactive compounds, especially ascorbic acid, the acerola fruits were transported under refrigeration (5 °C) on the same day of collection and immediately processed. After washing with water, the fruits were frozen in an ultra-freezer at − 80 °C (Coldlab CL580-86 V) and subsequently lyophilized (Lyophilizer L108, Liotop). Freeze-drying was selected because it enables water removal while minimizing the degradation of thermolabile bioactive compounds. Then the samples were crushed in a processor (Philips Walita RI7625 / 71) for 5 min to obtain a powder. The powder was stored at -80 °C until analysis.

For extract preparation, samples were mixed with water and ethanol solutions at a 1:60 (m/v) ratio and subjected to extraction in a shaking water bath at 200 rpm under low light conditions for 50 min, a duration previously established for the recovery of antioxidant compounds from acerola using hydroethanolic solvents [5]. The extraction conditions, including ethanol concentration and temperature ranges, were selected based on previous studies investigating the recovery of antioxidant compounds from acerola and other fruit matrices using hydroethanolic solvents. Ethanol and water mixtures have been widely reported as efficient green solvents for the simultaneous extraction of ascorbic acid and phenolic compounds. In addition, the evaluated temperature range was selected to investigate its influence on extraction efficiency while avoiding excessive degradation of thermosensitive compounds [4, 5]. The temperature in the extraction and concentration of the added ethanolic solution varied according to the experimental design. After the extraction period, the extracts were centrifuged at 5,000 × *g *for 20 min, filtered, and analyzed directly in liquid form for RSM response evaluation.

Extraction conditions were optimized by multiple regression analysis and response surface methodology, using reducing power as the response variable. A second-order model including linear, quadratic, and interaction terms was fitted for solvent concentration and temperature. The experimental design comprised five levels for each variable, including lower (− 1), upper (+ 1), and axial (± 1.41) points (Table 1). The aqueous extract obtained under the optimized RSM conditions was filtered through a 0.45 μm cellulose ester membrane (Millipore, 47 mm diameter) under vacuum, frozen at − 80 °C, lyophilized (Lyophilizer L108, Liotop), and stored at − 80 °C until further analysis.

**Table 1:** Reducing power, TEAC ABTS, and TEAC DPPH of hydroethanolic extracts from green acerola fruits at different extraction conditions

Run	Exploratory variables				Dependent variable		
	% Ethanol		Temperature ºC		Reducing power (mg GA g⁻¹ DM)	TEAC DPPH (µmol Trolox 100 mL^− 1^)	TEAC ABTS (µmol Trolox 100 mL^− 1^)
	Coded value	Real value	Coded value	Real value			
1	-1	14.4	-1	34.4	167.62	822.28	1170.91
2	+ 1	84.6	-1	34.4	150.91	808.00	997.89
3	-1	14.4	+ 1	55.6	159.74	786.50	982.02
4	+ 1	84.6	+ 1	55.6	155.08	843.42	1218.45
5	-1.41	0	0	45	160.05	575.42	1423.61
6	+ 1.41	99	0	45	151.03	611.42	1295.75
7	0	49.5	-1.41	30	164.69	820.57	1179.16
8	0	49.5	+ 1.41	60	152.25	801.71	1216.23
9	0	49.5	0	45	162.17	826.85	1626.15
10	0	49.5	0	45	159.23	799.42	1667.50
11	0	49.5	0	45	148.50	789.14	1625.07
12	0	49.5	0	45	156.56	851.42	1589.32

Values are single determinations per experimental run. Central point replicates (runs 9–12) used for pure error estimation

### Determination of Reducing Power

The reducing power for the acerola extracts was determined by the Folin-Ciocalteau colorimetric method according to the methodology proposed by Singleton et al. [9]. The results were calculated from a standard curve with known concentrations (5 to 80 µg∙mL^− 1^) of gallic acid and expressed in mg of Gallic acid (GA) per gram of dry fruit.

### Free Radical Scavenging Capacity (ABTS)

The free radical scavenging activity determined by the free radical ABTS (2,2’-azino-bis(3-ethylbenzothiazoline-6-sulphonic acid), was done according to the method described by Al-Duais et al [10]. The results were calculated from a standard curve with known concentrations (from 0.01 to 0.04 µmol∙mL^− 1^) of Trolox and expressed as the Trolox equivalent antioxidant capacity (µmol Trolox·100 mL⁻¹ of extract).

### Free Radical Scavenging Capacity (DPPH)

The free radical scavenging activity determined by the free radical DPPH (2,2-diphenyl-1-picrylhydrazyl) was done according to the method of Al-Duais et al [10]. The results were calculated from a standard curve with known concentrations (from 0.01 to 0.10 µmol∙mL^− 1^) of Trolox and expressed as the Trolox equivalent antioxidant capacity (µmol Trolox·100 mL⁻¹ of extract).

### Quantification of Ascorbic Acid

Quantification of ascorbic acid was performed using high-performance liquid chromatography (HPLC) according to the method described by Cruz et al [5]. A standard curve was done for ascorbic acid range from 100 to 1500 mg∙L^− 1^. The chromatography was performed on an Agilent HP series 1100 (series UV/Vis), with a diode array detector (DAD), a quaternary pump and a UV detector with a wavelength of multiple waves (MWD), a cooled column compartment (30 °C), and a C_18_ Phenomenex^®^ column (4,6 × 250 mm–5 μm). The mobile phase consisted of a gradient of citrate sodium solution (0.01 M) and methanol, started with 100% of citrate sodium and ending with 100% of methanol in 25 min. The flow rate was 1 mL∙min^− 1^, the injection volume was 20 µL. The DAD was set to collect the signal at 275 nm.

### Antioxidant Activity Using Yeast Cells

*Saccharomyces cerevisiae* strain BY4742 (Wild Type) and the *erg6*Δ mutant (EUROSCARF, Frankfurt, Germany) were used in this study. Subcultures of yeasts were performed by placing one isolated colony in 250 mL conical flasks containing 100 mL of Yeast extract-Peptone-Dextrose (YPD) medium. Flasks were placed in a rotary shaker Excella E24 (New Brunswick Scientific TM, Edison, NJ, USA) at 250 rpm, 25 °C for 48 h. Cultures were performed by transferring 1 ml of subcultures into a flask containing 100 ml of fresh YPD medium and cells were grown at 25 °C during 24 h in a rotary shaker at 250 rpm. At harvest, cells were in the beginning of the stationary phase, and the cell concentration was approximately 10^8^ cells∙mL^− 1^. Cell suspension (20 mL) was centrifuged for 5 min at 2.800×*g* and 25 °C (Eppendorf, Hamburg, Germany, model 5810R). Cell pellet was washed twice and after that the optical density (OD) was adjusted in 0.5 (600 nm, GE Healthcare^®^ GeneQuant 1300 spectrophotometer) in phosphate buffered solution (PBS).

To measure the antioxidant activity of the green acerola extract, the cells were subjected to oxidative stress conditions. Menadione (0.4 mM) was added to the cell suspension adjusted to an OD of 0.5 and incubated for 1, 3, 5, and 7 h. After each exposure time, the cells were centrifuged for 10 min at 5,000 × *g* and 25 °C and washed twice with PBS. Viable cells (CFU, Colony-Forming Units) were enumerated on YPD agar by spread plating followed by incubation at 25 °C for 48 h. Green acerola extract (500 mg∙L⁻¹) was added simultaneously with menadione. In addition, the same procedure was performed using cells maintained under the same conditions without oxidative stress and using cells treated only with green acerola extract (500 mg∙L⁻¹) to evaluate whether the extract itself could influence cell growth and viability. The concentration of 500 mg L⁻¹ of green acerola extract was selected based on the study by Cruz et al [5] in which different concentrations of acerola extracts were evaluated in Saccharomyces cerevisiae under different oxidative stress conditions. According to the authors, this concentration provided a significant protective effect against oxidative damage without causing deleterious effects on cell viability in the absence of oxidative stress.

### Spray Drying

Arabic gum (Sigma, 6 g *per* 100 mL of extract) was added as wall material to the optimized aqueous extract and stirred for 3 h until complete solubilization, following Tonon et al [11]. The resulting feed solution was spray-dried using a Mini Spray Dryer B-290 (BÜCHI Labortechnik AG, Flawil, Switzerland) equipped with a 0.7 mm two-fluid nozzle and a cyclone collector. Operating conditions were inlet temperature 180 ± 2 °C, outlet temperature 79 ± 2 °C, feed rate 8 g·min⁻¹, atomization gas flow 667 L·h⁻¹, and drying gas flow 35 m³·h⁻¹. The powder was collected, vacuum-packed, and stored at − 80 °C until analysis. To evaluate the effect of spray drying on the chemical profile of the extract, the powder was reconstituted in water at the original extract concentration under agitation (200 rpm, 3 h). Reducing power, ABTS, DPPH, and ascorbic acid content were then determined as described above. Water activity was measured at 25 °C using an AquaLab model 3TE (Decagon Devices, USA).

### Statistical Analysis

Non-significant terms (*p* > 0.05) were excluded by backward elimination before fitting the final response surface models. Model adequacy was assessed by the coefficient of determination (R²), regression significance, and lack-of-fit test through analysis of variance using Protimiza software (confidence level 95%). All remaining assays were performed in triplicate and results expressed as mean values. One-way ANOVA followed by Tukey’s *post-hoc* test was applied for pairwise comparisons, with *p* < 0.05 as the significance threshold (R software).

## Results and Discussion

Reducing power and antioxidant activity were selected as response surface methodology (RSM) responses, as both are widely recognized as indicators of ascorbic acid content in fruits with high concentrations such as green acerola [5, 12–14]. Ethanol and water were selected as solvents given their low toxicity and suitability for food-grade applications, and temperature was evaluated between 30 and 60 °C, the lower limit reflecting typical ambient conditions in Brazilian processing facilities and the upper limit set to minimize energy costs and thermal degradation of bioactive compounds. The experimental design, extraction conditions, and corresponding antioxidant responses for all 12 runs are presented in Table 1.

**Fig. 1:**
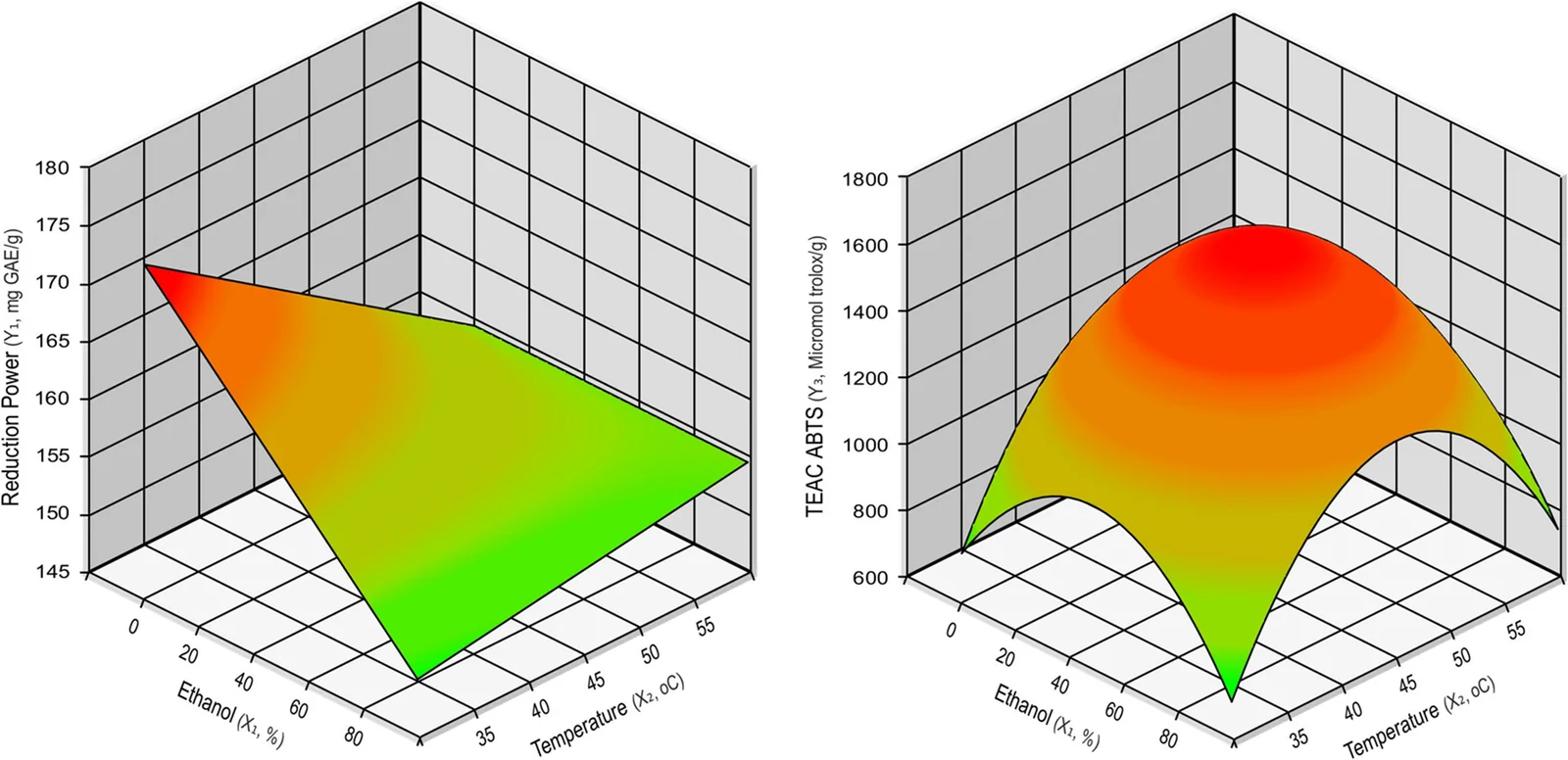
Response surfaces for (**a**) reducing power (mg gallic acid g⁻¹ dry matter) and (**b**) TEAC ABTS (µmol Trolox g⁻¹) as a function of ethanol concentration and temperature in green acerola fruit extracts

Reducing power varied by 9.97% across the experimental conditions, reflecting the influence of both solvent composition and temperature on the extraction of antioxidant compounds. Such variation is consistent with the literature, where RSM studies with different plant matrices report a wide range of antioxidant responses, reinforcing the complexity of food matrices and the need for matrix-specific optimization [15–18]. RSM enabled systematic evaluation of these variables and their interactions, providing a robust framework for identifying optimal extraction conditions with a reduced number of experiments [12]. Effect analysis indicated that linear and quadratic ethanol concentration terms were significant for reducing power, as was the interaction between ethanol concentration and temperature (*p*<0.05). For DPPH antioxidant activity, only the linear and quadratic ethanol concentration terms were significant, whereas both temperature and ethanol concentration showed significant linear and quadratic effects on ABTS activity. Non-significant terms were excluded, and second-order models were fitted (Table 2), evaluated by ANOVA (Table 3), and used to identify optimal extraction conditions. Response surfaces are shown in Fig. 1.

**Table 2:** Second-order polynomial models fitted for reducing power and antioxidant activity (TEAC ABTS and TEAC DPPH) of green acerola extracts

Response	Second-order polynomial equation (coded variables)
Reducing power (mg GA g⁻¹ DM)	*Ŷ*₁ = 158.15 − 4.27 *X*₁ − 2.66 *X*₁² + 3.01 *X*₁*X*₂
TEAC DPPH (µmol Trolox·100 mL^− 1^)	*Ŷ*₂ = 837.05 + 11.65 *X*₁ − 88.52 *X*₁²
TEAC ABTS (µmol Trolox·100 mL^− 1^)	*Ŷ*₃ = 1627.01 − 14.68 *X*₂ − 180.26 *X*₂² + 10.51 *X*₁ − 261.25 *X*₁²

*X*₁: ethanol concentration (%); *X*₂: temperature (°C); DM: dry matter; GA: gallic acid

**Table 3:** Analysis of variance (ANOVA) for the response surface models fitted to green acerola extraction data

Source of variation	Sum of squares	DF	Mean square	F value	*p*-value
Regression	238.6016	3	79.5339	9.4524	0.0052
Residue	67.3132	8	8.4141	1.8850	0.3192
Lack of fit	51.06070	5	10.2121		
Pure error	16.2525	3	5.4175		
Total	305.9148	11			

R² = 78.00% (coefficient of determination)

The R² of 78.00% refers to the reducing power model (Ŷ₁); TEAC DPPH and TEAC ABTS models were fitted independently (Tables 2 and 3). The moderate fit reflects the narrow variation range across conditions (9.97%) and could be improved by incorporating additional variables in future studies.

Both ethanol concentration and temperature negatively affected reducing power, with pure water at the lowest temperatures yielding the highest values. Notably, ethanol solutions between 30 and 60% recovered higher amounts of other antioxidant compounds, indicating a matrix-dependent solvent effect. Based on these results, two conditions were selected for validation in triplicate using the same fruit lots: pure water and 40% ethanol, both at 35 °C (Table 4). The choice of 35 °C rather than 30 °C was supported by the absence of statistically significant differences in reducing power between these temperatures, with the higher value adopted to provide greater operational robustness under food-grade processing conditions. Further studies assessing additional parameters, such as extraction time, as well as alternative extraction techniques, including ultrasound-assisted extraction, could provide further improvements in extraction efficiency and promote the recovery of even higher concentrations of bioactive compounds of interest.

**Table 4:** Reducing power, ascorbic acid content, and antioxidant activity (TEAC ABTS and TEAC DPPH) of aqueous and hydroethanolic extracts of green acerola under optimized conditions

Samples	Reducing power (mg GA g⁻¹ DM)	TEAC DPPH (µmol Trolox g⁻¹ DM)	TEAC ABTS (µmol Trolox g⁻¹ DM)	Ascorbic acid (mg g⁻¹ DM)
Water extract	201.40^a^	1067.15^a^	1232.94^a^	340.12^a^
Ethanol extract	191.40^b^	1045.24^a^	1111.99^a^	285.68^b^

*GA* gallic acid. Results expressed per gram of dry matter (DM). Values followed by different superscript letters within the same column indicate significant differences (*p* < 0.05, Tukey’s test, *n* = 3)

Ascorbic acid content was significantly higher in aqueous extracts (*p* < 0.05), confirming the strong correlation between reducing power and ascorbic acid in acerola and supporting the mathematical model predictions. Aqueous extracts presented reducing power and TEAC values among the highest reported for tropical fruits, including species widely recognized as antioxidant sources such as jaboticaba and camu-camu [19, 20]. Consequently, all subsequent analyses were performed with aqueous extracts. Dry matter content of the optimized aqueous extract was 8.860 mg·mL⁻¹, of which 63.98% corresponded to ascorbic acid. These values exceed those previously reported for green acerola by Vendramini and Trugo [21], who used water and dichloromethane without extraction optimization, which likely accounts for the lower recovery observed in that study. While not investigated in the present work, previous studies have shown that hydroethanolic extraction can enhance the recovery of non-ascorbic antioxidant compounds from acerola, including quercetin, chlorogenic acid, and p-coumaric acid. These phenolic compounds may also contribute to reducing power and TEAC values, helping to explain the improved recovery of antioxidant constituents observed in hydroethanolic extracts [5, 21].

**Fig. 2:**
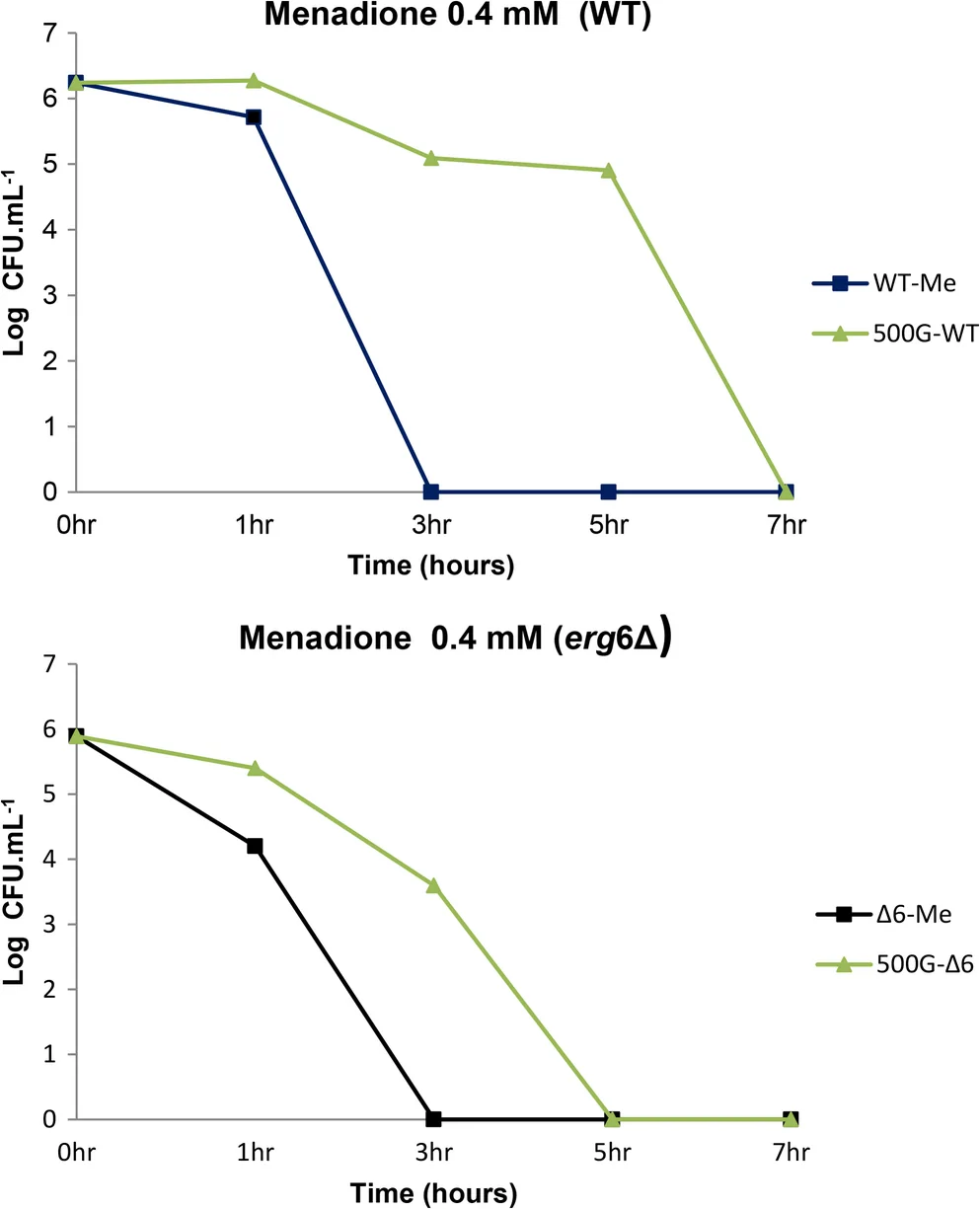
Cell viability (log CFU·mL⁻¹) of Saccharomyces cerevisiae wild-type (WT) and erg6Δ (Δ6) strains exposed to 0.4 mM menadione over time in the presence (G: menadione + green acerola extract, 500 mg·L⁻¹) or absence (Me: menadione only) of the extract. Control groups included C (only yeast cells) and 500G-(WT or Δ6)-C (yeast cells with green acerola extract, 500 mg·L⁻¹)

Although in vitro antioxidant assays are widely used, they do not reflect cellular conditions, and complementary in vivo or cell-based data are necessary to evaluate the biological relevance and safety of new ingredients [22]. *Saccharomyces cerevisiae* represents a well-established cellular model for this purpose, given its genetic similarity to higher eukaryotes, ease of handling, high reproducibility, and low cost [23]. Wild-type (WT) and *erg6*Δ mutant strains were used to assess cell cultivability under menadione-induced oxidative stress. The *erg6*Δ mutant was selected due to its heightened sensitivity to oxidative stress, attributed to its inability to synthesize ergosterol [24, 25]. Menadione promotes oxidative damage in *S. cerevisiae* by depleting intracellular glutathione through conjugate formation and export, thereby reducing cellular resistance to oxidative reactions [26]. Results for both strains over time are shown in Fig. 2.

Green acerola extract alone did not affect yeast cell growth or viability. As shown in the graph, the control group containing only cells maintained a similar number of viable cells throughout the experiment when compared to cells exposed exclusively to green acerola extract (500 mg L⁻¹). These results indicate that the extract did not exert either toxic or growth-promoting effects under the experimental conditions, confirming that the differences observed under oxidative stress were associated with its protective antioxidant activity rather than with changes in cell proliferation.

The green acerola extracts significantly preserved cell viability under menadione-induced oxidative stress. Given the high ascorbic acid content of the optimized aqueous extract (63.98% of dry matter, Table 4), it is the primary candidate responsible for the antioxidant protection observed. However, co-extracted phenolic constituents present in the aqueous fraction, including flavonoids and hydroxycinnamic acids previously identified in acerola matrices [7], may also contribute synergistically to the cellular protective effect.

In the wild-type strain, cultivability was maintained for up to 5 h in the presence of the extract (500 mg L⁻¹), compared to approximately 1 h in the menadione-only control. The *erg6*Δ mutant showed lower overall resistance, with colony counts declining more rapidly from the first hour; however, the presence of acerola extracts extended cultivability to 3 h, compared to complete loss of viability in its absence. These findings are consistent with the lower oxidative resistance attributed to the *erg6*Δ strain due to its inability to synthesize ergosterol, a membrane sterol with a recognized antioxidant role [24, 27].

Since menadione induces oxidative stress primarily through the intracellular generation of superoxide anions [5], and the acerola extracts were added simultaneously with the oxidant, the protective effect observed is likely associated, at least in part, with ROS-scavenging activity. The fact that protection was detected in both the wild-type and erg6Δ strains supports an antioxidant action of the extracts. Nevertheless, the greater susceptibility of the erg6Δ strain indicates that membrane sterol composition also contributes to oxidative stress tolerance. Therefore, the protective effect observed may result from a combination of ROS scavenging and membrane-related mechanisms, although the relative contribution of each cannot be determined from the present data.

Liquid fruit extracts are susceptible to microbiological deterioration and chemical instability, particularly when rich in sensitive bioactive compounds. Spray drying offers an effective stabilization strategy, converting liquid extracts into powders with reduced water activity, lower storage costs, and higher compound concentration [28, 29]. The spray-dried green acerola powder presented a water activity of 0.213 at 25 °C, well below the 0.6 threshold for microbial stability, confirming microbiological safety under the processing conditions applied. Despite the thermal stress inherent to spray drying, no significant changes were observed in any of the analyzed parameters before and after the process (Table 5), demonstrating effective preservation of the antioxidant profile.

**Table 5:** Effect of spray drying on the chemical profile of green acerola extract

	Green acerola extract (100 mg)	Spray-dried green acerola extract (100 mg)
Reducing power (mg gallic acid)	25.50^a^	22.94^a^
TEAC DPPH (µmol Trolox)	259.82^a^	257.97^a^
TEAC ABTS (µmol Trolox)	220.20^a^	216.19^a^
Ascorbic acid (mg)	43.09^a^	40.66^a^

Values followed by different superscript letters within the same column indicate significant differences (*p* < 0.05, Tukey's test, *n* =3)

Preservation of the antioxidant profile after spray drying is consistent with the literature. Saenz et al. [30] reported up to 100% recovery of betacyanins and indicaxanthines from cactus extract microencapsulated with arabic gum, with no reduction in antioxidant activity, under conditions comparable to those applied in the present study. Similarly, Krishnaiah et al [31] reviewed the protective effect of carrier materials on bioactive compounds during spray drying of fruit extracts, supporting the suitability of arabic gum as a wall material for ascorbic acid-rich matrices. Despite the absence of statistically significant differences, the slight reductions observed in ascorbic acid content and reducing power may reflect both analytical variability and limited thermal degradation of heat-sensitive compounds, particularly ascorbic acid. However, the small magnitude of these changes suggests that the spray-drying conditions employed, together with the protective effect of arabic gum, were effective in preserving the antioxidant profile of the extract.

The nutritional relevance of the powder is noteworthy: based on the FAO [32] recommended daily intake of 45 mg vitamin C for adults, the requirement would be met by only 0.77 g of the spray-dried powder, which also delivers additional antioxidant compounds. Growing restrictions on synthetic antioxidants in food and pharmaceutical products, driven by concerns over their potential adverse health effects, have increased demand for natural alternatives with consumer acceptance [33, 34]. In this context, the green acerola powder presents promising potential as a clean-label natural antioxidant ingredient across multiple product categories. Furthermore, the use of pure water at mild temperature as the sole extraction solvent directly addresses one of the main environmental drawbacks of current industrial vitamin C production, which relies on organic solvents and energy-intensive chemical steps [3]. Taken together, these results demonstrate that pure water extraction at mild temperature, validated by both in vitro and cellular antioxidant assays and combined with spray drying stabilization, constitutes a complete and environmentally favorable processing chain for converting green acerola into a stable, vitamin C-rich ingredient. The elimination of organic solvents at the extraction stage, the demonstrated biological activity in a eukaryotic cellular model, and the preservation of antioxidant properties after spray drying collectively support the potential of this approach for natural food ingredient development.

## Conclusions

Response surface methodology identified pure water at mild temperature as the optimal extraction condition, maximizing ascorbic acid recovery and antioxidant activity from green acerola fruits while eliminating the need for organic solvents. The biological relevance of the optimized extract was confirmed using a *Saccharomyces cerevisiae* cellular model, in which cell viability was preserved under oxidative stress for up to 5 h in the wild-type strain and 3 h in the *erg6*Δ mutant, compared to approximately 1 h in untreated controls. The differential response between strains reinforces the role of membrane ergosterol in cellular antioxidant protection. Spray drying with arabic gum as carrier produced a stable powder with water activity of 0.213, with no significant loss of antioxidant composition or activity. These findings establish green acerola as a promising natural source of vitamin C and antioxidant compounds for food ingredient applications, obtained through an environmentally favorable process suitable for further scale-up investigation. 

## Data Availability

The data supporting the findings of this study are available from the corresponding author upon reasonable request.

## Abbreviations and Nomenclature

GA: Gallic acid
ABTS: azinobis (3-ethylbenzothiazoline-6-sulfonic acid)
DPPH: 1,1-diphenyl-2-picrylhydrazyl radical
TEAC: Trolox equivalent antioxidant capacity
WT: Wild Type
OD: Optical Density
PBS: Phosphate buffered saline
CFU: Colony-forming Unit
RSM: Response Surface Methodology

## References

[1] Svirbely J, Szent-Györgyi A (1932) The chemical nature of vitamin C. Biochem J 26:865–870. 10.1042/bj0260865 PMC1260981 16744896

[2] Piro A, Tagarelli G, Lagonia P, Tagarelli A, Quattrone A (2010) Casimir Funk: his discovery of the vitamins and their deficiency disorders. Ann Nutr Metab 57:85–88. 10.1159/000319165 20805686

[3] Pappenberger G, Hohmann H-P (2014) Industrial production of l-ascorbic acid (vitamin C) and d-isoascorbic acid. In: Zorn H, Czermak P (eds) Biotechnology of food and feed additives. Springer, Berlin, pp 143–188. 10.1007/10_2013_243 24258144

[4] Guzman NO, Pizzaro RA (2025) Bioactive compounds from tropical fruit by-products: extraction, characterization and therapeutic potential. J Agric Food Res 21:101983. 10.1016/j.jafr.2025.101983

[5] Cruz RG, Beney L, Gervais P, Lira SP, Vieira TMFS, Dupont S (2019) Comparison of the antioxidant property of acerola extracts with synthetic antioxidants using an in vivo method with yeasts. Food Chem 277:698–705. 10.1016/j.foodchem.2018.10.099 30502205

[6] Oledzki R, Harasym J (2024) Acerola (Malpighia emarginata) anti-inflammatory activity—a review. Int J Mol Sci 25:2089. 10.3390/ijms25042089 PMC10889565 38396766

[7] Vilvert JC, de Freitas ST, dos Santos LF, Ribeiro TS, Veloso CM (2024) Phenolic compounds in acerola fruit and by-products: an overview on identification, quantification, influencing factors, and biological properties. J Food Meas Charact 18:216–239. 10.1007/s11694-023-02175-1

[8] Melo SKS, Carvalho AJBA, Costa MM, Vale RB, Silva JA, Alencar MG, Barros AC, Queiroz MAA, Panea B, Rosa DS, Carvalho FAL (2026) Active packaging with microencapsulated concentrates of acerola (Malpighia emarginata) from different stages of maturity in the shelf life of lamb meat. Eur Food Res Technol 252:25. 10.1007/s00217-025-04986-6

[9] Singleton VL, Orthofer R, Lamuela RM (1999) Analysis of total phenols and other oxidation substrates and antioxidants by means of Folin-Ciocalteau reagent. Methods Enzymol 299:152–178. 10.1016/S0076-6879(99)99017-1

[10] Al-Duais M, Muller L, Bohm V, Jetschke G (2009) Antioxidant capacity and total phenolics of Cyphostemma digitatum before and after processing: use of different assays. Eur Food Res Technol 228:813–821. 10.1007/s00217-008-0994-8

[11] Tonon RV, Brabet C, Hubinger MD (2010) Anthocyanin stability and antioxidant activity of spray-dried açai (Euterpe oleracea Mart.) juice produced with different carrier agents. Food Res Int 43:907–914. 10.1016/j.foodres.2009.12.013

[12] Subramani V, Tomer V, Balamurali G, Mansingh P (2025) Artificial neural network in optimization of bioactive compound extraction: recent trends and performance comparison with response surface methodology. Anal Sci 41:101–117. 10.1007/s44211-024-00681-w 39503809

[13] Pérez M, Domínguez-López I, Lamuela-Raventós RM (2023) Total phenolic intake in a Mediterranean dietary pattern. J Agric Food Chem 71:17543–17553. 10.1021/acs.jafc.3c04022 PMC10682990 37948650

[14] Zugazua-Ganado M, Bordagaray A, Ezenarro J, García-Arrona R, Ostra M, Vidal M (2024) Adaptation of the Folin–Ciocalteu and Fast Blue BB spectrophotometric methods to digital image analysis for the determination of total phenolic content: reduction of reaction time, interferences and sample analysis. LWT Food Sci Technol 193:115756. 10.1016/j.lwt.2024.115756

[15] Weremfo A, Abassah-Oppong S, Adulley F, Dabie K, Seidu-Larry S (2022) Response surface methodology as a tool to optimize the extraction of bioactive compounds from plant sources. J Sci Food Agric 103:26–36. 10.1002/jsfa.12121 35833361

[16] Athanasiadis V, Kachrimanidou V, Koutinas AA, Dimitrellou D (2023) Optimization of extraction parameters for enhanced recovery of bioactive compounds from quince (Cydonia oblonga) peel waste. Foods 12:2099. 10.3390/foods12112099 PMC10252653 37297343

[17] Maccarronello AE, Cardullo N, Silva AM, Di Francesco A, Costa PC, Rodrigues F, Muccilli V (2024) From waste to bioactive compounds: a response surface methodology approach to extract antioxidants from Pistacia vera shells for postprandial hyperglycaemia management. Food Chem 443:138504. 10.1016/j.foodchem.2024.138504 38309024

[18] Ozakn G, Ugur ES, Capanoglu E (2026) Ultrasonication enabled liposomal encapsulation of propolis extract: bioaccessibility, bioavailability, and food application. Food Bioprocess Technol 19:6–16. 10.1007/s11947-026-04205-4

[19] Rufino MSM, Alves RE, Brito ES, Pérez-Jiménez J, Saura-Calixto F, Mancini-Filho J (2010) Bioactive compounds and antioxidant capacities of 18 non-traditional tropical fruits from Brazil. Food Chem 121:996–1002. 10.1016/j.foodchem.2010.01.037

[20] Danielski R, Shahidi F (2024) Nutraceutical potential of underutilized tropical fruits and their byproducts: phenolic profile, antioxidant capacity, and biological activity of jerivá (Syagrus romanzoffiana) and butiá (Butia catarinensis). J Agric Food Chem 72:4035–4048. 10.1021/acs.jafc.3c06350 38349961

[21] Vendramini AL, Trugo LC (2000) Chemical composition of acerola fruit (Malpighia punicifolia L.) at three stages of maturity. Food Chem 71:195–198. 10.1016/S0308-8146(00)00152-7

[22] Silva CG, Raulino RJ, Cerqueira DM, Mannarino SC, Pereira MD, Panek AD, Silva JFM, Menezes FS, Eleutherio ECA (2009) In vitro and in vivo determination of antioxidant activity and mode of action of isoquercitrin and Hyptis fasciculata. Phytomedicine 16:761–767. 10.1016/j.phymed.2008.12.019 19200698

[23] Assalve G, Lunetti P, Zara V, Ferramosca A (2024) In vivo antioxidant activity of common dietary flavonoids: insights from the yeast model Saccharomyces cerevisiae. Antioxidants 13:1103. 10.3390/antiox13091103 PMC11429029 39334762

[24] Dupont S, Lemetais G, Ferreira T, Cayot P, Gervais P, Beney L (2012) Ergosterol biosynthesis: a fungal pathway for life on land? Evolution 66:2961–2968. 10.1111/j.1558-5646.2012.01667.x 22946816

[25] Dupont S, Fleurat-Lessard P, Cruz RG, Lafarge C, Grangeteau C, Yahou F, Gerbeau-Pissot P, Abrahão Júnior O, Gervais P, Simon-Plas F, Cayot P, Beney L (2021) Antioxidant properties of ergosterol and its role in yeast resistance to oxidation. Antioxidants 10:1024. 10.3390/antiox10071024 PMC8300696 34202105

[26] Zadziliski R, Fortuniak AZ, Bilifiski T, Grey M, Bartosz G (1998) Menadione toxicity in Saccharomyces cerevisiae cells: activation by conjugation with glutathione. Biochem Mol Biol Int 44:747–759. 10.1080/15216549800201792 9584988

[27] Dupont S, Beney L, Ferreira T, Gervais P (2011) Nature of sterols affects plasma membrane behavior and yeast survival during dehydration. Biochim Biophys Acta 1808:1520–1528. 10.1016/j.bbamem.2010.11.012 21081111

[28] Díaz-Montes E (2023) Wall materials for encapsulating bioactive compounds via spray-drying: a review. Polymers 15:2659. 10.3390/polym15122659 PMC10303636 37376305

[29] Feihrmann AC, Silva NM, Marins AR, Matiucci MA, Nunes KC, Nakamura CV, Souza MLR, de Oliveira O, Gomes RG (2024) Ultrasound-assisted extraction and encapsulation by spray drying of bioactive compounds from Tradescantia zebrina leaves. Food Chem Adv 4:100621. 10.1016/j.focha.2024.100621

[30] Saenz C, Tapia S, Chavez J, Robert P (2009) Microencapsulation by spray drying of bioactive compounds from cactus pear (Opuntia ficus-indica). Food Chem 114:616–622. 10.1016/j.foodchem.2008.09.095

[31] Krishnaiah D, Nithyanandam R, Sarbatly R (2014) A critical review on the spray drying of fruit extract: effect of additives on physicochemical properties. Crit Rev Food Sci Nutr 54:449–473. 10.1080/10408398.2011.587038 24236997

[32] FAO/WHO (2001) Human vitamin and mineral requirements: Report of a joint FAO/WHO expert consultation. Food and Agriculture Organization of the United Nations

[33] Sousa G, Trifunovska M, Antunes M, Miranda I, Moldão M, Alves V, Vidrih R, Lopes PA, Aparicio L, Neves M, Tecelão C, Ferreira-Dias S (2021) Optimization of ultrasound-assisted extraction of bioactive compounds from Pelvetia canaliculata to sunflower oil. Foods 10:1732. 10.3390/foods10081732 PMC8391403 34441510

[34] Novais C, Molina AK, Abreu RMV, Santo-Buelga C, Ferreira ICFR, Pereira C, Barros L (2022) Natural food colorants and preservatives: a review, a demand, and a challenge. J Agric Food Chem 70:2783–2805. 10.1021/acs.jafc.1c07533 PMC9776543 35201759

